# Targeting cancer stem cells as a strategy for reducing chemotherapy resistance in head and neck cancers

**DOI:** 10.1007/s00432-023-05136-9

**Published:** 2023-07-15

**Authors:** Dawid Dorna, Jarosław Paluszczak

**Affiliations:** Department of Pharmaceutical Biochemistry, Poznan University of Medical Sciences, Ul. Święcickiego 4, 60-781 Poznan, Poland

**Keywords:** Cancer stem cells, Chemosensitivity, Chemotherapy resistance, Cisplatin, CSC, Head and neck cancer

## Abstract

**Purpose:**

Resistance to chemotherapy and radiotherapy is the primary cause of a poor prognosis in oncological patients. Researchers identified many possible mechanisms involved in gaining a therapy-resistant phenotype by cancer cells, including alterations in intracellular drug accumulation, detoxification, and enhanced DNA damage repair. All these features are characteristic of stem cells, making them the major culprit of chemoresistance. This paper reviews the most recent evidence regarding the association between the stemness phenotype and chemoresistance in head and neck cancers. It also investigates the impact of pharmacologically targeting cancer stem cell populations in this subset of malignancies.

**Methods:**

This narrative review was prepared based on the search of the PubMed database for relevant papers.

**Results:**

Head and neck cancer cells belonging to the stem cell population are distinguished by the high expression of certain surface proteins (e.g., CD10, CD44, CD133), pluripotency-related transcription factors (SOX2, OCT4, NANOG), and increased activity of aldehyde dehydrogenase (ALDH). Chemotherapy itself increases the percentage of stem-like cells. Importantly, the intratumor heterogeneity of stem cell subpopulations reflects cell plasticity which has great importance for chemoresistance induction.

**Conclusions:**

Evidence points to the advantage of combining classical chemotherapeutics with stemness modulators thanks to the joint targeting of the bulk of proliferating tumor cells and chemoresistant cancer stem cells, which could cause recurrence.

## Introduction

Head and neck squamous cell carcinomas (HNSCC) are the most common type of neoplastic lesions that develop in the head and neck region. With over 900,000 new cases and over 450,000 deaths in 2020, HNSCC is the eighth most common cancer worldwide (Sung et al. 2021). The incidence of HNSCC continues to rise and is expected to increase by 30% by 2030 (Ferlay et al. 2019). Many patients are diagnosed at an advanced stage of the disease. Thus, they often do not have good long-term prognosis. Like other cancer types, head and neck cancers are managed by surgery, radiotherapy, and chemotherapy (Atashi et al. 2021). Due to the localization of lesions, surgical resection often causes permanent disfigurement and a decrease in quality of life. As a result, survivors of this cancer have the second highest rate of suicide when compared to survivors of other cancers (Osazuwa-Peters et al. 2018). The current standard of care for patients with locally advanced HNSCC is concomitant platinum-based chemoradiotherapy (CRT) or surgery followed by adjuvant radiation or chemoradiation. For patients with recurrent and/or metastatic HNSCC, platinum-based chemotherapy plus 5-fluorouracil (5-FU) has a response rate of 30–40% and a median survival of 6–9 months. Patients with platinum-resistant disease have few options and a very poor prognosis with second-line therapies (Sola et al. 2019). Thus, novel therapeutic strategies augmenting the effects of treatment could significantly benefit HNSCC patients. Furthermore, due to the frequent resistance to conventional treatment, extensive research has been conducted to develop molecularly targeted therapies. So far, only cetuximab, an epidermal growth factor receptor (EGFR) inhibitor, and, more recently, nivolumab and pembrolizumab, PD-1 inhibitors, have been approved for the treatment of HNSCC. However, cetuximab, a monoclonal antibody approved by the FDA in 2006, shows only limited efficacy in advanced HNSCC patients (Sola et al. 2019).

Many factors that contribute to resistance to therapy in HNSCC can be identified. The most studied mechanisms involve alterations in intracellular drug accumulation, detoxification, and DNA damage repair in cancer cells. Other novel mechanisms include epigenetic changes that regulate cell plasticity, the involvement of the tumor microenvironment (TME), and the presence of so-called cancer stem cells (CSCs) (Griso et al. 2022). CSCs constitute a small cell population characterized by slow proliferation, self-renewal capacity by symmetric or asymmetric division, and resistance to therapy (Atashzar et al. 2020; Yang et al. 2020). It is believed that CSCs may be derived from transformed adult stem cells, or they can originate by the dedifferentiation of somatic cells (Barbato et al. 2019; Walcher et al. 2020; Yin et al. 2021). A recent hypothesis states that adult stem cells are the cell population that is most likely to accumulate oncogenic mutations and serve as cancer cells of origin (White and Lowry 2015). The biological behavior of CSCs is determined by the action of several pluripotency and self-renewal-mediating transcription factors, including c-MYC, Nanog, OCT-3/4, SOX2, KLF4, and by the activity of stemness-related signaling pathways, typically Wnt/β-catenin, Hedgehog, Notch, JAK/STAT, TGF-β/SMAD, PI3K/Akt, and NF-kappaB, together with intercellular and extracellular matrix (ECM) communication within the TME niche (Huang et al. 2020a, b; Yang et al. 2020).

CSCs tend to be radio- and chemoresistant, which results from several mechanisms: (1) the upregulated expression of the ABC family of transporters, which are responsible for the exclusion of cytotoxic drugs from cancer cells; (2) the induction of quiescence/dormancy; (3) the enhancement of DNA repair mechanisms; (4) increased protection against oxidative stress; (5) cell plasticity (Barbato et al. 2019; Gupta et al. 2021; Kuşoğlu and Biray Avcı 2019; Yang et al. 2020; Yin et al. 2021). While traditional chemotherapy kills the rapidly dividing cells that constitute the majority of the tumor mass, CSCs may remain intact and cause cancer relapse after the end of treatment (Atashzar et al. 2020; Barbato et al. 2019; Kuşoğlu and Biray Avcı 2019; Walcher et al. 2020). Indeed, chemoresistant cells or cancer stem cells form slow-growing, aggressive/metastatic tumors in mice (Mir et al. 2021). Eliminating CSCs to improve therapy response is not a novel idea, and the scientific community has been exploring these options for decades (Atashzar et al. 2020; Barbato et al. 2019; Gupta et al. 2021; Walcher et al. 2020; Yang et al. 2020). However, this appears to be challenging (Griso et al. 2022).

CSCs in solid tumors are identified by the presence of various cell surface (CD10, CD24, CD44, CD90, CD133, CD271, EpCAM, LGR5) or intracellular (ALDH1, Nanog, OCT3/4, BMI-1, SOX2) markers (Kuşoğlu and Biray Avcı 2019; Walcher et al. 2020). HNSCC cells belonging to the CSC population are distinguished by their high expression of certain surface proteins (for example, CD44, CD133), pluripotency-related transcription factors (SOX2, OCT4, Nanog), and increased aldehyde dehydrogenase (ALDH) activity (Cirillo et al. 2021; Krishnamurthy et al. 2010; Prince et al. 2007). The major markers of HNSCC stem cells are presented in Table 1. Prince et al. (2007) were the first to report the existence of a population of neoplastic cells with stem cell properties in HNSCC (Prince et al. 2007). They observed that the CD44 + cell population could form new tumors in vivo, in contrast to the CD44 – cell population. Moreover, CD44 + cells were less differentiated than CD44 – cells, which more closely resembled a differentiated squamous epithelium and showed increased involucrin expression (a marker of keratinocyte differentiation). Tumors that arose from the isolated population of CD44 + cells recreated the heterogeneity of the primary tumor and could be passaged multiple times, which proved that the CD44 + population had two key stem cell features—the ability for differentiation and self-renewal. Krishnamurthy et al. (2010) showed that, based on the assessment of ALDH activity within the CD44 + tumor cell population, it is possible to identify a subpopulation of cells with an even greater intensity of features typical of stem cells, which constituted 1–3% of the cells of the primary tumor (Krishnamurthy et al. 2010). In their studies, the implantation of 1000 CD44 + /ALDH + cells led to the formation of tumors in 13 out of 15 mice, while the implantation of 10,000 CD44 –/ALDH – cells led to the development of tumors in only two animals. ALDH1 activity has also been associated with an increased frequency of local relapse after the end of therapy (Ota et al. 2014). Later studies have shown that these stem-like cells are also much less sensitive to chemoradiotherapy and can persist after therapy, leading to relapse. The overexpression of CD44 correlated with poor overall and disease-free survival in patients with advanced oral carcinomas (Boxberg et al. 2018). In addition, the expression of ALDH1 and CD44 was a predictor of angiolymphatic invasion and lymph node metastasis in patients with oral carcinomas, respectively (Ortiz et al. 2018). In another study, ALDH1 expression was associated with lymph node involvement and high mortality rate (Gupta et al. 2022). A broader stem cell gene expression signature correlated with lower 5-years and relapse-free survival rates in HPV-negative HNSCC patients (Kim et al. 2022). HNSCC tumors developing in Fanconi anemia patients carry a very poor prognosis and require aggressive treatment. Notably, these tumors contain a greater proportion of ALDH-positive CSC cells showing Nanog and Oct-3/4 expression, in comparison with sporadic HNSCC (Wu et al. 2014). Thus, current evidence shows that an increase in cancer stem cell population confers poor prognosis in HNSCC.

**Table 1:** The major markers of the cancer stem cell subpopulations in HNSCC

Protein	Physiological function	Relation to HNSCC stem-like phenotype
CD10	Membrane metallopeptidase responsible for the cleavage of signaling peptides. Involved in cell differentiation and growth (Fukusumi et al. 2014)	CD10 + cells were slow-cycling and resistant to cisplatin, 5-fluorouracil, and radiation. They expressed higher levels of ALDH1, OCT4, BMI1, and NANOG and formed more tumorospheres (Fukusumi et al. 2014; Pu et al. 2021; Wang et al. 2021)
CD44	Hyaluronic acid receptor, involved in cell adhesion and migration. Links the extracellular matrix and pluripotency-related transcription factors like SOX2, OCT4, and Nanog (Bourguignon et al. 2012; Cirillo et al. 2021)	CD44 + cells exhibited decreased proliferation but higher colony formation ability and were more resistant to cisplatin and EGFR inhibition. They were also less differentiated and more tumorigenic than CD44 − cells (La Fleur et al. 2012; Prince et al. 2007)
CD133	Cell surface protein, widely distributed on protrusions of plasma membranes with possible involvement in membrane structure organization (Wu and Wu 2009)	Side population cells expressed CD133 and exhibited high self-renewal capacity. These cells were resistant to cisplatin, oxaliplatin, paclitaxel, and 5-fluorouracil, which was associated with increased expression of ABCG2 and Bcl2 (Guan et al. 2015; Lu et al. 2016)
CD271	Receptor for neurotrophin and nerve growth factor, involved in survival, differentiation and migration of neuronal cells (Murillo-Sauca et al. 2014)	CD44 + CD271 + cells exhibited high tumorigenic potential, enhanced capacity for tumorsphere formation, and increased expression of BMI1, OCT4, SOX2, NANOG, and drug efflux transporters, as well as showed increased resistance to cisplatin and 5-fluorouracil (Elkashty et al. 2020; Imai et al. 2013; Murillo-Sauca et al. 2014)
SOX2, OCT4, NANOG	Transcription factors highly active in embryonic stem cells and involved in maintaining their self-renewal ability and in cell fate determination (Cirillo et al. 2021)	Associated with self-renewal ability, frequently overexpressed in chemoresistant and highly tumorigenic HNSCC cell populations (Elkashty et al. 2020; Imai et al. 2013; Keysar et al. 2017; Mishra et al. 2020; Pu et al. 2021; Wang et al. 2021)
BMI1	Transcriptional repressor, member of the Polycomb family, involved in chromatin structure regulation and stem cell renewal (Chen et al. 2017)	BMI1 + cells exhibited high clonogenic potential, were highly tumorigenic in vivo and contributed to cisplatin resistance. These cells were able to transit from slowly proliferating to rapidly dividing cells (Chen et al. 2017)
ALDH1	An enzyme involved in aldehyde detoxification and oxidation of vitamin A precursor, which is linked with the regulation of cell growth and differentiation (Yu and Cirillo 2020)	ALDH + cells showed higher expression of BMI1, OCT4, SOX2, KLF4, and NANOG, and reduced radiosensitivity. CD44 + cells with high ALDH activity were more tumorigenic (Chen et al. 2010; Gunduz et al. 2019; Krishnamurthy et al. 2010)
ABCG2	A drug efflux protein from the ABC transporters family (Cirillo et al. 2021)	Highly expressed in side population cells, simultaneously with other stemness markers like CD44, CD133, and ALDH1A (Jiang et al. 2018; Sinnung et al. 2021)

Abbreviations: *CD10* Cluster of Differentiation 10, *CD44* Cluster of Differentiation 44, *CD133* Prominin-1 or Cluster of Differentiation 133, *CD271* Low-affinity Nerve Growth Factor Receptor or Cluster of Differentiation 271, *SOX2* SRY-Box Transcription Factor 2, *OCT4* Octamer-Binding Transcription Factor 4, *NANOG* Homeobox Protein NANOG, *BMI1* Polycomb Group Ring Finger 4, *ALDH1* Aldehyde Dehydrogenase 1 Family, Member A1, *ABCG2* ATP Binding Cassette Subfamily G Member 2

The growing field of research on the importance of cancer stem cells in HNSCC has resulted in many new and interesting findings in recent years. This narrative review aimed to present the latest evidence documenting the significance of cancer stem cells in the development of therapeutic resistance in head and neck squamous cell cancers, including the molecular mechanisms involved in the stemness-related development of resistance. We searched the PubMed database using the keywords “head neck cancer stem cell resistance” and retrieved records from the last decade (Jan 2012–Feb 2023). We identified additional records by cross-references. Our analysis included all the experimental papers that tested the association between stemness potential and resistance. Most studies have focused on the chemoresistance to conventional chemotherapeutics, mainly cisplatin, but also 5-fluorouracil or docetaxel. We excluded papers that merely reported an association between resistance phenotype and the expression of stemness markers, and we focused on papers that reported evidence generated with the use of stem cell subpopulations. Aiming to focus on squamous cell carcinomas, we also excluded papers studying esophageal, thyroid, salivary gland, nasopharyngeal or central nervous system tumors, because of different etiological and clinical factors related to these tumor types. Additionally, we excluded experimental papers that used the misidentified Hep2 cell line as a model of HNSCC.

Several review papers have recently been published on this topic (Cirillo et al. 2021; Mudra et al. 2021; Siqueira et al. 2023), but new information has appeared since. This review presents up-to-date knowledge and focuses on the possibilities of pharmacological targeting of stemness-related chemoresistance. More information about the relationship between stemness and radioresistance can be found in other excellent reviews (Atashzar et al. 2020; Siqueira et al. 2023).

## The association between stemness phenotype and chemoresistance in HNSCC

### Chemotherapy leads to the enrichment of cancer stem cells

Several lines of evidence point to the appearance of cancer stem cells as the driving force in chemoresistance. First of all, many studies have shown that chemotherapy and radiotherapy can increase the percentage of cancer stem cells. In this regard, cisplatin led to an increase in the percentage of ALDH-positive cells (Kim et al. 2017; Subramanian et al. 2017) or CD44^high^ cells (Basak et al. 2015; Bu et al. 2015), or ALDH^high^CD44^high^ cells (Nakano et al. 2021; Nör et al. 2014). Cells identified as the side population (SP) exhibit the ability to efflux the Hoechst33342 dye, which is a measure of drug efflux capability reflecting cellular chemoresistance. To a large extent, these cell populations overlap with stem-like cells (Yang et al. 2020). Cisplatin has been shown to increase the ratio of CD44 + cells or the percentage of SP cells, which show elevated expression of CD44, CD133, ALDH1A, and ABCG2 (Jiang et al. 2018). In addition, cisplatin increased the SP and CD24 + cell populations (Sinnung et al. 2021). Moreover, ionizing radiation increases the percentage of side population cells (Macha et al. 2017). Thus, in general, chemoradiotherapy leads to a dangerous enrichment with CSCs, which poses a significant risk of treatment failure in the long term (Dzobo et al. 2020). It remains unclear whether this is solely a consequence of killing the bulk of proliferating cancer cells or whether chemotherapeutics can transform cells toward a stem-like phenotype. Although this is difficult to discern experimentally, some evidence points to the possibility of the latter (Nör et al. 2014; Vipparthi et al. 2022). Moreover, stem cell plasticity may be responsible for the adaptive response to chemotherapy, leading to resistance (Gupta et al. 2021). HNSCC cell lines and tumor-derived cells exhibit different stem cell subpopulations based on the presence of CD44, CD24, and ALDH markers. CD44^high^ cells may transition into CD44^high^ALDH^high^ cells or CD44^high^CD24^high^ cells, and the latter could also gain ALDH activity. It has been observed that while the CD24 transition was unidirectional, there was plasticity/reversibility on the ALDH axis. Notably, the acquisition of cisplatin resistance was related to stem cell phenotype switching. Cisplatin induced the transition toward CD24^high^ cells and stimulated plasticity toward the ALDH^high^ subpopulation. Indeed, triple-positive cells (CD44^high^CD24^high^ALDH^high^) were the most enriched subpopulation after cisplatin treatment, presenting a highly cisplatin-tolerant phenotype associated with high expression of ABCG2 drug efflux protein (Vipparthi et al. 2022).

Many studies focused on cancer stem cells used the tumorsphere assay to evaluate the effects on chemosensitivity. This assay is a simple measure of stemness-associated self-renewal under low-attachment conditions and is utilized to enrich the subpopulation of cancer stem cells (Yang et al. 2020). Cisplatin was shown to increase the efficiency of sphere formation in the tumorsphere assay (Nör et al. 2014; Subramanian et al. 2017). Indeed, cells grown as spheres showed an increased level of CD44, SOX2, OCT4, NANOG, and c-Myc, compared to monolayer cells (Huang et al. 2020a, b). SAS cells grown as spheres were less sensitive to cisplatin or gemcitabine than parental cells (Sun et al. 2022a, b). Moreover, stem cells isolated from SAS cells orospheres were much less sensitive to cisplatin than parental cells (Peng et al. 2022). Additionally, HNSCC stem cells generated by growing parental cells as spheres for three generations showed elevated expression of ALDH1, SOX2, and KLF4 and lowered sensitivity to cisplatin or 5-fluorouracil (Garcia-Mayea et al. 2019). Thus, there is a clear association between the exposure to cytostatic drugs and cancer stem cells accummulation, either because of their selection following the killing of bulk cancer cells, or due to stimulation of cell plasticity, or both.

### Drug-resistant cells show the enrichment in stem cell subpopulations

Another line of evidence comes from studies using resistant cell lines generated in vitro by prolonged sequential treatment of cells with increasing drug concentrations. Such resistant cells exhibit enhanced stemness compared to the parental cells. For example, cisplatin-resistant CAL27 and SCC-131 cells were able to form larger tumorspheres, pointing to higher self-renewal potential, and exhibited elevated expression of CD44, KLF4, OCT4, SOX2, c-MYC, and β-catenin (Roy et al. 2018, 2019). Similarly, cisplatin-resistant OC2 cells showed a greater capacity for tumorsphere formation and increased expression of CD133, ABCG2, BMI1, OCT4, and NANOG (Tsai et al. 2011). Multidrug (cisplatin, docetaxel, doxorubicin, erlotinib) resistant HSC-3 cells showed higher expression of CD44 and SOX9 and increased ability for tumorsphere formation (Murakami et al. 2022). Furthermore, CAL27 cells resistant to cisplatin or docetaxel were enriched in CD44 + cells and showed elevated expression of CD133, ALDH1A1, OCT4, SOX2 (Kulsum et al. 2017), and cisplatin-resistant CAL27, and SCC9 cells showed the accumulation of CD44 + ALDH + cells (Lima de Oliveira et al. 2022). In addition, cisplatin-resistant FaDu cells showed increased expression of CD44 and an increased percentage of CD44-positive cells. They also exhibited increased autophagy, which inhibition with anti-ATG14 siRNA reduced CD44 expression (Naik et al. 2018). On the other hand, cisplatin-resistant Detroit 562 cells are enriched in CD10-positive cells (Fukusumi et al. 2014). Cisplatin-resistant SAS cells showed higher ALDH activity and increased expression of CD133, OCT4, and NANOG (C.-W. Chang et al. 2014). Similarly, cisplatin-resistant UM-SCC-22B cells exhibited higher expression of BMI1 and OCT4 pluripotency markers (Nör et al. 2014). Cisplatin-resistant SCC-4/-9 cells showed elevated expression of NANOG, which transcriptionally stimulated OCT4, c-MYC, and ABCG2 expression, which was reduced by NANOG knockdown, leading to sensitization to cisplatin (Kashyap et al. 2020). Moreover, immunohistochemical analysis showed the upregulation of OCT4 and NANOG in OSCC patients characterized by chemoresistance, which indicates that these in vitro findings have clinical relevance (Tsai et al. 2011). Thus, it can be concluded that chemoresistant cells are characterized by the accumulation of cancer stem cells and the increased expression of stemness (CD44, CD133, CD10, ALDH) and pluripotency (NANOG, BMI1, OCT4, SOX2) markers. Thus, the acquisition of cellular chemoresistance is indeed associated with increased stemness potential.

### Isolated cancer stem cells are resistant to chemotherapeutics

The most compelling evidence for the key importance of targeting cancer stem cells in tackling chemoresistance comes from studies that used the subpopulations of CSCs isolated by selective cell sorting based on the presence of stem cell markers. Since no single marker of HNSCC stem cells exists, studies focused on analyzing different stemness-related proteins, with CD44 and ALDH being the most frequently investigated. For example, ALDH + cells showed higher expression of BMI1, OCT4, NANOG, and MDR1 and reduced radiosensitivity (Chen et al. 2010). In another study, a small subpopulation of UTSCC-60A cells that were positive for ALDH expressed higher levels of BMI1, KLF4, OCT4, and SOX2 and were resistant to cisplatin (Gunduz et al. 2019). In parallel, ALDH^low^ cells were more sensitive to paclitaxel (Fernandes et al. 2022). ALDH-positive cells are usually described as a subpopulation of CD44^high^ cells, and these cells were resistant to docetaxel or cetuximab (Keysar et al. 2017). Moreover, a subpopulation of CD44v3^high^ALDH^high^ HNSCC stem cells, which expressed OCT4, NANOG, and SOX2, was resistant to apoptosis induction because of the high expression of IAP proteins (XIAP, c-IAP2). The presence of hyaluronic acid (HA), which interacts with CD44, further decreases cisplatin-induced apoptosis, whereas anti-CD44 antibody sensitizes cells to cisplatin (Bourguignon et al. 2012, 2016). This underscores the role of the HA-CD44 axis in HNSCC chemoresistance. Histopathological analyses seem to corroborate these findings because the increased immunohistochemical levels of ALDH1, CD44, or pSTAT3 were associated with shorter overall survival in HNSCC patients, while the worst survival rate was observed in triple-positive patients (Chen et al. 2010). Additionally, CD44^high^ cells showed lower proliferation but higher colony formation ability and were resistant to cisplatin, and tended to be resistant to EGFR inhibition by cetuximab or gefitinib (La Fleur et al. 2012). CD44 + HNSCC cells were resistant to apoptosis induction, and showed elevated expression of anti-apoptotic Bcl-2 and IAP proteins (Chikamatsu et al. 2012). The ratio of CD44 + cells significantly varies among different HNSCC cell lines and not all CD44 + cells exhibit stem-like properties and chemoresistance (Modur et al. 2016). Furthermore, subpopulations of CD44^high^ cells were distinguished based on differences in cell morphology, and ameboid-like CD44^high^ cells showed significant resistance to docetaxel, compared to epithelial-like or mesenchymal-like CD44^high^ OSCC cells (Yokoyama et al. 2021). In another study, mesenchymal-like CD44^high^ cells, which appeared when cells were grown on fibronectin-coated hydrogel, were characterized by elevated expression of NANOG, SOX2, OCT4, and ALDH1 and resistance to cisplatin when compared to epithelial-like CD44^high^ or parental cells (Shigeishi et al. 2022). Thus, it is relevant to recognize that CD44^high^ cells are neither homogeneous nor a fixed population of cells. Indeed, stemness seems to depend on cell plasticity and constitutes the feature of the tumor as a whole (Wang et al. 2017). On the other hand, in vitro growth conditions affect stem cells by inducing adaptive changes due to cell plasticity. It remains to be established which culture conditions are best in mimicking the in vivo environments to allow the best possible prediction of therapeutic response.

CD271 belongs to the TNF family of receptors, and it is present in the stem cells of the normal oral epithelium. However, its growing expression has been observed in various stages of pathology: from dysplasia to advanced HNSCC cases (Elkashty et al. 2020). Interestingly, the presence of CD271 is restricted to CD44 + cells and CD44 + CD271 + cells turned out to have the highest tumorigenic potential (Murillo-Sauca et al. 2014). Furthermore, CD44 + CD271 + cells exhibited a higher capacity for tumorsphere formation and increased expression of BMI1, OCT4, and SOX2 and showed resistance to cisplatin and, to a lesser extent, 5-fluorouracil (Elkashty et al. 2020). In an interesting study using patient-derived xenotransplanted hypopharyngeal tumors, cell sorting led to the identification of a subpopulation of tumor-initiating cells that were positive for CD271. These cells would tend to be located in tumors at the invasive front and near blood vessels. They were also highly tumorigenic in mice. Moreover, CD271 + cells expressed NANOG, OCT4, and SOX2 and cell surface efflux transporters, e.g., ABCC2, ABCB5, and ABCG2. Importantly, cisplatin was able to kill CD271-negative cells while CD271-positive cells survived cisplatin treatment in vivo. This suggests that the presence of CD271 marks a subpopulation of cisplatin-resistant HNSCC stem cells (Imai et al. 2013).

Although rarely detected in HNSCC samples (Fukusumi et al. 2014), CD133 is yet another marker of stem cells. A small percentage of HNSCC-derived cells were characterized as SP cells which expressed stemness markers (CD133 and OCT4) and showed high self-renewal capacity. These cells were resistant to cisplatin, oxaliplatin, paclitaxel, and 5-fluorouracil, which was associated with increased expression of ABCG2 drug efflux transporter and anti-apoptotic Bcl2 (Guan et al. 2015; Lu et al. 2016). In another study, CD44^high^CD133^high^CD117^high^ HN13 cells were much less sensitive to paclitaxel treatment than parental cells (viability 88% vs 44%, respectively) (Silva Galbiatti-Dias et al. 2018).

Moreover, several studies have shown that HNSCC stem cells also exhibit the presence of CD10 or CD24 surface markers. Indeed, CD10 + cells formed more tumorspheres, expressed higher levels of ALDH1 and OCT3/4, and were tumorigenic in mice. These slow-cycling dormant cells were resistant to cisplatin, 5-fluorouracil, and radiation (Fukusumi et al. 2014). Additionally, CD10^high^ cells showed higher expression of ALDH1, BMI1, OCT4, NANOG, and SOX2 and were significantly less sensitive to cisplatin, than CD10^low^ cells (Pu et al. 2021; Wang et al. 2021). In addition, higher expression of CD24, NANOG, and OCT4 correlated with a reduced response to cisplatin combined with radiotherapy in patients with OSCC (Mishra et al. 2020). Furthermore, the percentage of CD24 + cells correlated with cisplatin resistance in HNSCC cell lines, and CD24 knockdown significantly reduced NANOG expression and sensitized cells to cisplatin treatment. Moreover, CD24 + cells were enriched in the fraction of residual resistant cells (Modur et al. 2016).

## The pharmacological targeting of stemness-mediated HNSCC chemoresistance mechanisms

**Fig. 1:**
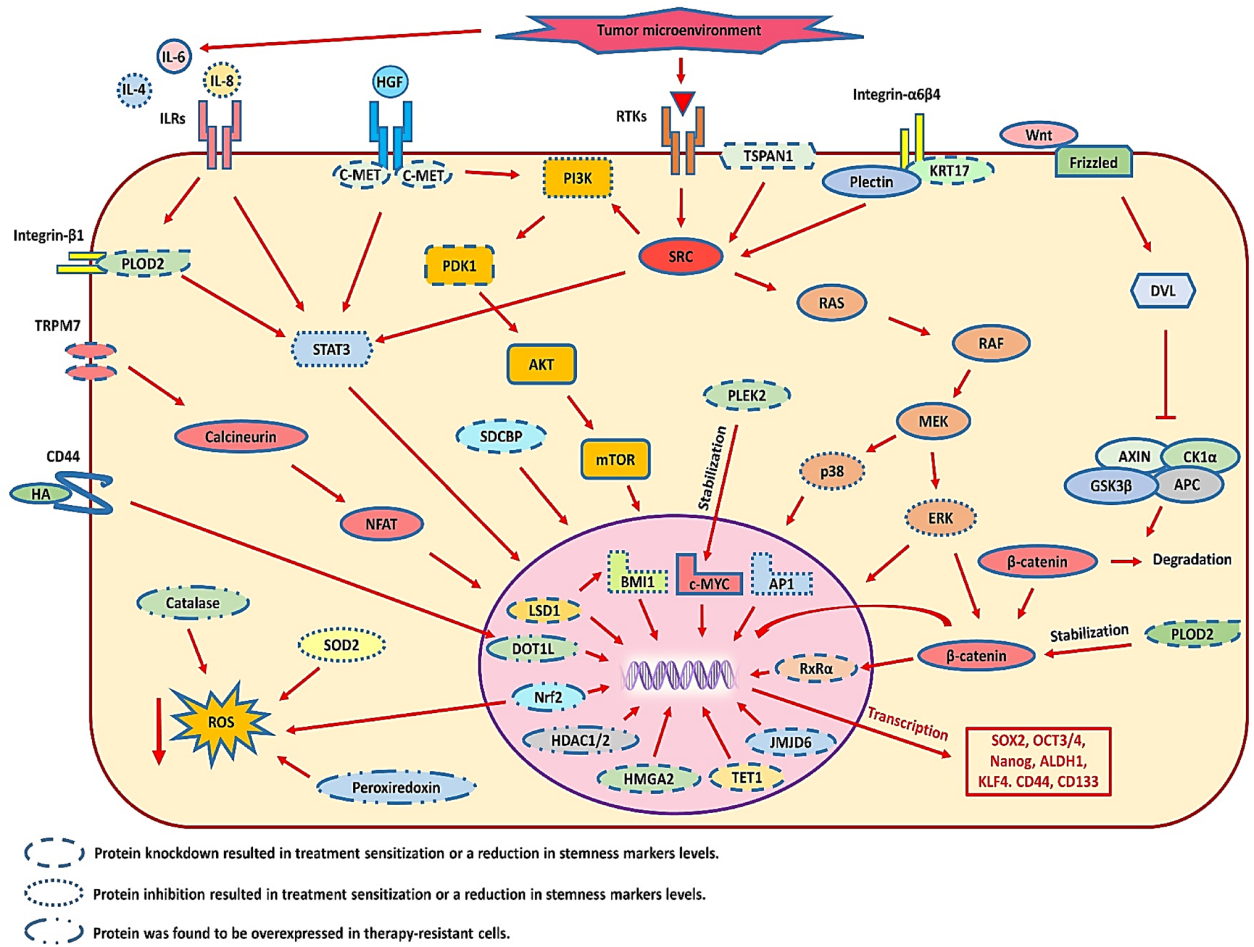
The molecular mechanisms responsible for stemness-associated resistance (Bu et al. 2015; Chen et al. 2017, 2022, 2010; Garcia-Mayea et al. 2020; Han et al. 2021; Herzog et al. 2021; Hsueh et al. 2021; Huang et al. 2020a, b; Jang et al. 2022; Jiang et al. 2018; Kashyap et al. 2018; Keysar et al. 2017; Lee et al. 2016; Lim et al. 2014; Paluszczak 2020; Peng et al. 2018; Silva Galbiatti-Dias et al. 2018; Sinnung et al. 2021; Song et al. 2022; Wang et al. 2017, 2018; Zhao et al. 2022). Abbreviations: *c-MET* tyrosine-protein kinase Met, *DOT1L* DOT1 like histone lysine methyltransferase, *HA* hyaluronic acid, *HDAC1/2* histone deacetylase 1/2, *HGF* hepatocyte growth factor, *HMGA2* high mobility group AT-hook 2, *IL-4,6,8* interleukin-4,6,8, *ILRs* interleukin receptors, *KRT17* keratin 17, *LSD1* lysine-specific demethylase 1, *NFAT* nuclear factor of activated T-cells, *PLEK2* pleckstrin 2, *PLOD2* procollagen lysine 2, *RTKs* receptor tyrosine kinases, *SRC* proto-oncogene tyrosine-protein kinase Src, *SDCBP* syntenin-1, *STAT3* signal transducer and activator of transcription 3, *TET1* tet methylcytosine dioxygenase 1, *TSPAN1* tetraspanin-1, *TRPM7* transient receptor potential cation channel, subfamily M, member 7

A complex network of molecular pathways regulates the transcriptional and cellular programs responsible for the stemness phenotype. Thus, many potential molecular targets (Fig. 1) could be therapeutically modulated to prevent relapse by facilitating the elimination of chemoresistant cancer stem cells and thus increasing the effectiveness of chemotherapeutics.

### PI3K/Akt, Wnt/β-catenin and Src pathways interactively induce stemness-related resistance

ALDH + CD44^high^ cells showed activation of the PI3K/Akt/mTORC1 pathway, which regulates SOX2 expression, in turn activating ALDH1A1 expression and activity. These cells responded to PI3K inhibition, which decreased the ALDH + population and SOX2 expression without affecting CD44 expression. Moreover, SOX2 overexpression diminished the response to docetaxel (Keysar et al. 2017). The Akt kinase activation can also be mediated by PDK1, whose knockdown reduced the level of pAkt, and affected stemness by reducing the expression of SOX2, OCT4, and CD133, decreasing tumorsphere formation capacity. Moreover, the PDK1 inhibitor—BX795—sensitized OSCC cells to cisplatin (Pai et al. 2021). Thus, the PI3K/Akt pathway is mechanistically responsible for the induction of ALDH and SOX2 expression and participates in stemness-related chemoresistance. These effects may be mediated by cross-talk with other signaling pathways. The canonical Wnt/β-catenin pathway has been implicated in HNSCC development (Paluszczak 2020), and a recent study has shown that pAkt stimulates β-catenin nuclear translocation, which induces the TCF4-mediated transcription of ALDH1A1 (Wang et al. 2017). Moreover, the activation of the Wnt/β-catenin pathway plays a role in the cisplatin-induced enrichment of resistant stem cells (Sinnung et al. 2021). Cisplatin-resistant and CSC cells showed the elevated level of β-catenin and EZH2. EZH2, which is a histone methyltransferase mediating transcriptional repression by H3K27 methylation, suppressed APC which acts as the upstream inhibitor of β-catenin activation. The combinatorial inhibition of both Wnt/β-catenin and EZH2 effectively reduced the CSC population in vitro and in vivo, and sensitized cells to cisplatin (Milan et al. 2023). Notably, the Wnt pathway also influences RXR-mediated effects. In this regard, the overexpression of RXRα or the addition of retinoids (9-cis-retinoic acid) led to the enrichment of SP cells and CD44 + cells, and increased the level of expression of stemness markers (CD44, CD133, SOX2, OCT4), while the knockdown of RXRα resulted in the opposite effects (Jiang et al. 2018).

On the other hand, a pathway initiated by the interaction of Keratin 17 (KRT17) with plectin and integrin-64 may stimulate the transcriptional activity of β-catenin. This pathway activates the FAK/Src/ERK cascade of downstream kinases, ultimately resulting in the nuclear translocation of β-catenin, leading to the upregulation of CD44 and enhanced sphere formation. Importantly, the knockdown of KRT17 reduced the self-renewal potential and sensitized cells to cisplatin (Jang et al. 2022). In addition, cisplatin-resistant cells expressed higher levels of tetraspanin-1 (TSPAN1), and its siRNA-mediated reduction enhanced susceptibility to cisplatin and dasatinib. Dasatinib is a small molecule inhibitor targeting the Src pathway. Indeed, TSPAN1 depletion reduced the level of active phospho-Src kinase, although TSPAN1 targeted both Src-dependent and independent pathways (Garcia-Mayea et al. 2020). Similarly, syntenin-1 (SDCBP) was upregulated in cisplatin-resistant and stem-like Detroit 562 cells, and the depletion of SDCBP sensitized the cells to cisplatin. This led to reduced expression of CD44, CD133, KLF4, and OCT3/4 and decreased levels of phospho-Src protein. Moreover, Src inhibition also sensitized cells to cisplatin (Mir et al. 2021). These data would suggest that Src may be an important downstream effector regulating stemness and chemoresistance in HNSCC. Indeed, the inhibitors of the Src family of kinases are an emerging group of anti-cancer molecularly targeted therapeutics. However, they did not demonstrate sufficient clinical effectiveness in HNSCC. In contrast to the aforementioned studies, a recent paper has shown that Src inhibitors—dasatinib or saracatinib—not only failed to eliminate cancer stem cells in tumorspheres but also increased the expression of ALDH1A1, SOX2, OCT4, and NANOG. The authors of this work hypothesized that this pro-stemness activity was responsible for the poor clinical response to these drugs. A mithramycin analog, EC-8042, on the other hand, reduced the stemness phenotype, and the combination of this compound with dasatinib was beneficial. Such simultaneous targeting of proliferating and migrating cells by dasatinib and tumor-propagating cells by EC-8042 led to potent antitumor activity in vivo (Hermida-Prado et al. 2019). Thus, the exact role of Src signaling in regulating stemness and pluripotency in HNSCC cells still requires elucidation.

The activation of the MEK/ERK pathway has been shown to contribute to cisplatin resistance by inducing the expression of CD44v4 and its pharmacological inhibition reversed this phenotype (Kashyap et al. 2018). In another study, the ERK1/2 pathway induced the expression of CD44 and NANOG and increased resistance to cisplatin or 5-fluorouracil in CD44 + cell spheroids. ERK inhibitors sensitized these cells to chemotherapeutics (Huang et al. 2020a, b). On the other hand, MEK/ERK inhibition did not affect ALDH expression (Keysar et al. 2017). The inhibition of p38 using SB203580 reduced the RNA and protein levels of stemness markers (CD44, OCT4, KLF4) in cisplatin-resistant SCC-131 and CAL27 cells. In addition, pretreatment of cells with the p38 inhibitor sensitized resistant cells to cisplatin, significantly increasing DNA damage and apoptosis. Moreover, SB203580 prevents cisplatin-induced enhancement of stemness marker expression (Roy et al. 2021).

### Interleukins induce stemness-related resistance via STAT3 activation

Several interleukins (IL) have been implicated in regulating stemness and resistance. Indeed, cisplatin-resistant HNSCC cells showed higher expression of IL-6/8/10 (Basak et al. 2015). Also, IL6 enhanced cisplatin-induced enrichment of the ALDH^high^CD44^high^ cells (Nör et al. 2014). The low level of let-7c in ALDH + and CD44 + cells was responsible for the upregulation of IL-8 secretion. Conversely, the overexpression of let-7c attenuated IL-8 level, reduced ALDH activity and sensitized ALDH + cells to cisplatin. However, the addition of IL-8 could antagonize these effects (Peng et al. 2018). Moreover, the increased secretion of IL-8 via ERK signaling activation enhanced chemoresistance in cisplatin-resistant CD10^high^ cells. IL-8 inhibition using SB225002 sensitized CD10^high^ cells to cisplatin (Pu et al. 2021). Additionally, it has been found that the Hedgehog signaling pathway is involved in regulating the cisplatin-resistant properties of CD10^high^ cells (Y. Wang et al. 2021). Also, the hypersecretion of IL-4 can drive the multidrug resistance phenotype of CD133 + side population cells and neutralizing IL-4 by antibody sensitized these cells to drug treatment (Guan et al. 2015). The cross-talk between JMJD6 and IL-4 further substantiates the importance of IL-4 for stemness. It has been shown that tumorsphere cells express higher levels of several histone demethylases, including JMJD6. Indeed, ALDH^high^ cells showed elevated expression of JMJD6. On the other hand, JMJD6 overexpression led to increased expression of stemness markers (OCT4, NANOG) and resulted in cell resistance to doxorubicin, etoposide, and methotrexate. Importantly, JMJD6 transcriptionally regulates IL-4. Anti-IL-4 antibodies suppressed the stem-like phenotype of JMJD6 overexpressing cells, while recombinant IL-4 rescued the stemness phenotype (C.-R. Lee et al. 2016). The association between immunomodulation, stemness, and chemoresistance is further supported by the observation that the upregulation of the CXCR3A chemokine receptor increased the expression of SOX2 and NANOG, and stimulated the resistance to cisplatin, gemcitabine, and paclitaxel (Sun et al. 2022a, b).

The activation of the STAT3 pathway, which can be mediated by IL-4, IL-6, or other factors, was shown to maintain the stemness potential of radioresistant ALDH + CD44 + cells. The decrease in phospho-STAT3 levels induced by cucurbitacin I stimulates the differentiation of these stem cells into ALDH/CD44-negative cells, sensitizing tumors to ionizing radiation (Chen et al. 2010). The activation of STAT3 correlated with the increased expression of ALDH1, CD44, OCT4, and SOX2 in cancer cells (Bu et al. 2015). Moreover, it has been shown that the activation of the IL-6/STAT3 pathway in cisplatin-resistant cell lines is driven by collagen lysyl hydroxylase PLOD2, which results in the stimulation of the expression of stemness markers CD44 and CD133 via integrin β1 (Song et al. 2022). PLOD2 can also activate Wnt signaling and PLOD2 overexpressing FaDu cells showed elevated expression of NANOG, OCT4, and KLF4. PLOD2 knockdown reduced the tumorsphere forming capacity, the percentage of SP cells, and sensitized cells to cisplatin treatment (Sheng et al. 2019). The silencing of IL-6R decreased the percentage of cisplatin-induced ALDH^high^CD44^high^ cells showing that IL-6/STAT3 signaling is important for the stemness phenotype by regulating the expression of BMI1 (Herzog et al. 2021). Magnololol-induced sensitization of orosphere-derived stem cells from the SAS cell line to cisplatin is mediated by the reduced secretion of IL-6 and decreased activation of STAT3. Moreover, magnolol attenuated ALDH activity and decreased the capacity for secondary sphere formation (Peng et al. 2022). The inhibition of STAT3 using S3I-201 led to the elimination of both bulk and side population cancer cells in vitro. Also, it diminished the capacity for tumorsphere formation and the expression of ALDH1, CD44, OCT4, and NANOG, resulting in the sensitization of cells to chemotherapeutics (Bu et al. 2015).

### Other players

TRPM7 is a membrane protein that functions as a channel for divalent cations (particularly Mg2 +) and contains a serine/threonine kinase domain. The protein acts as a sensor of changes in cellular osmolarity, and pH alterations. It has pleiotropic functions, and affects cell survival, proliferation and migration. It has been shown that cisplatin-resistant patients show higher RNA expression of TRPM7. The downregulation of this membrane receptor protein was associated with a decrease in the expression of stemness markers (BMI1, OCT4, SOX2, NANOG) in SAS cells. The knockdown of TRPM7 in combination with cisplatin strongly reduced the capacity for tumorsphere formation. These findings suggest that the TRPM7/NFAT pathway is relevant for maintaining OSCC stem cells (Chen et al. 2022).

Pleckstrin-2 (PLEK2) is another protein that is implicated in the regulation of stemness. Pleckstrin-2 is a cell membrane-associated protein which takes part in focal adhesion and contact with the actin cytoskeleton, and is also implicated in PI3K signaling. PLEK2 was found overexpressed in dysplasia and HNSCC, showing the highest expression in chemoresistant patients. Also, chemoresistant cell lines expressed higher levels of PLEK2. The overexpression of PLEK2 increased the proportion of ALDH + cells, while the knockdown of PLEK2 reduced the expression of stemness markers (CD133, BMI1, SOX2, OCT4, NANOG) and decreased ALDH activity. These effects were mediated by the stabilization and activation of c-MYC by PLEK2 (Zhao et al. 2022).

The HGF/c-MET pathway also plays a role in the maintenance of stemness phenotype and chemoresistance. Indeed, ALDH^high^ cells showed high expression of c-Met, while c-Met^high^ cells were characterized by the increased expression of OCT4, SOX2, and CD44. The knockdown of c-Met decreased the expression of these stem cell markers and diminished tumorsphere forming capacity. Moreover, it led to a decrease in the percentage of SP cells and reduced the expression of ABCG2 transporter protein, which was associated with modest cisplatin sensitization (Lim et al. 2014).

BMI1 is one of the pluripotency markers whose enhanced expression seems to play an important role in HNSCC cell stemness. CD44 + ALDH^high^ cells isolated from parental and cisplatin-resistant SCC-1 cells showed elevated expression of BMI1. Also, tumor-derived EpCAM + CD44 + ALDH^high^ cells showed elevated BMI1 expression. Indeed, BMI1 + cells were found to be slowly proliferating but could transform into actively proliferating cells, which points to their stem-like features. BMI1 + cells isolated from primary tumors showed high clonogenic potential, as shown by the ability to form primary and secondary tumorspheres. Also, they were highly tumorigenic in vivo, in contrast to BMI1 non-expressing cells. BMI1 has been associated with chemoresistance since PTC-209, an inhibitor of BMI1, restored the sensitivity of cisplatin-resistant SCC-1 cells to cisplatin. This points to the possible clinical potential of combining classical chemotherapeutics with stemness modulators thanks to the joint targeting of the bulk of proliferating tumor cells and chemoresistant cancer stem cells. Monotherapy with cisplatin killed mitotic cells and induced apoptosis of BMI-negative cells while enriching BMI + cells that were present in recurrent or persistent tumors in the mouse 4NQO-induced tumor model. This shows that the lack of elimination of cancer stem cells is responsible for treatment failure. Importantly, the combination therapy with cisplatin and PTC-209 effectively inhibited tumor growth. PTC-209 significantly decreased the percentage of BMI + cancer stem cells and its combination with cisplatin reduced both BMI1 + and bulk cancer cells in vivo. Importantly, a similar effect was observed with the AP-1 inhibitor, 3-PA, which underscores the importance of the AP1 pathway in regulating BMI1 expression (Chen et al. 2017).

CAL27 and FaDu cells grown as tumorspheres showed elevated expression levels of CD133, CD44, ALDH1, SOX2, and BMI1. The higher expression of these stemness markers may depend on the activity of the HMGA2 protein, which acts in cooperation with Slug. Knockdown of HMGA2 reduced the expression of CD133, CD44, ALDH1, SOX2, and BMI1, and diminished tumorsphere formation capacity. On the other hand, overexpression of HMGA2 increased tumorsphere formation and facilitated cell survival in the presence of cisplatin, thus causing chemoresistance (Li et al. 2022). Chaperone proteins are another player in therapy resistance. The pharmacological inhibition of Heat shock protein 90 (Hsp90) with KU711 or KU757 decreased the number of spheres and the percentage of ALDH-positive and CD44-positive cells and the level of BMI1 protein in parental and cisplatin-resistant HNSCC cell lines (Subramanian et al. 2017).

### Epigenetic regulation of transcription and CSC chemoresistance

Epigenetic mechanisms can affect transcriptional programs associated with cell plasticity and the induction of the expression of pluripotency and stemness-related genes. For example, CD44 may lead to chemoresistance by increasing the expression of anti-apoptotic IAP proteins. These effects are mediated by the upregulation of DOT1L histone lysine methyltransferase and the subsequent increase in the methylation level of H3K79 residue, which directs the activation of gene transcription (Bourguignon et al. 2016). The observation that the knockdown of TET1 protein, which is responsible for active DNA demethylation, may sensitize CD44 + cells to cisplatin further supports the association between stemness-related chemoresistance and epigenetic mechanisms. TET1 promotes chemoresistance by MGMT promoter demethylation, augmenting DNA repair response to damages induced by alkylating agents (Wang et al. 2018). Moreover, cisplatin-resistant cell lines showed the overexpression of histone deacetylases HDAC1/2 (Lima de Oliveira et al. 2022). On the other hand, histone lysine demethylase LSD1 was essential for the stimulation of the expression of BMI1. LSD1 knockdown suppressed stemness characteristics, although it led to the upregulation of PDL1, enhancing immune evasion. However, the combination of LSD1 inhibition and PD-1 blockade showed efficacy in vivo, leading to overcoming immune evasion (Han et al. 2021).

### Redox states and CSC chemoresistance

Stemness and resistance are also associated with redox homeostasis. For instance, cisplatin was found to elevate the proportion of stem-like ROS^low^ cells. Cisplatin-resistant SAS cells exhibited low levels of reactive oxygen species (ROS) due to increased expression and activity of catalase, superoxide dismutase 2 (SOD2), or peroxiredoxin. Thus, the depletion of ROS scavengers may stimulate chemosensitivity. Indeed, cell treatment with 2-metoxyestradiol and/or 3-amino-1,2,4-triazole lowered the expression of OCT4 and NANOG and reduced the proportion of ROS^low^ cells, thus sensitizing cells to cisplatin (Chang et al. 2014). Interestingly, FaDu cells that acquired resistance to PI3K inhibitor BEZ235 and cross-resistance to gefitinib and cisplatin exhibited stemness phenotype. Specifically, these cells had elevated activity of ALDH and increased expression of NANOG, OCT4, SOX2, and BMI1 but also displayed ROS imbalance and SOD2 upregulation. Notably, SOD inhibitors sensitized these resistant cells to BEZ235 (Hsueh et al. 2021). Also, cisplatin-resistant CAL27 and SCC9 cells demonstrated reduced ROS levels. The inhibition of HDAC6 by tubastatin A induced oxidative stress in these cells, reversing the cisplatin-induced accumulation of CD44^high^ALDH^high^ stem cells (Tavares et al. 2022). Moreover, increasing ROS formation by the inhibition of ALDH activity with Aldi-6 contributed to cell sensitization to cisplatin, which could be counteracted by the addition of antioxidant *N*-acetylcysteine (Kim et al. 2017). Additionally, the chemoresistant CD133 + side population cells exhibited increased expression of the Nrf2 transcription factor, which promotes the expression of cytoprotective and antioxidant proteins (Lu et al. 2016). These findings indicate the significant contribution of redox imbalance in the acquisition and/or maintenance of chemoresistance in stem-like HNSCC cancer cells.

### The important role of tumor microenvironment

**Fig. 2:**
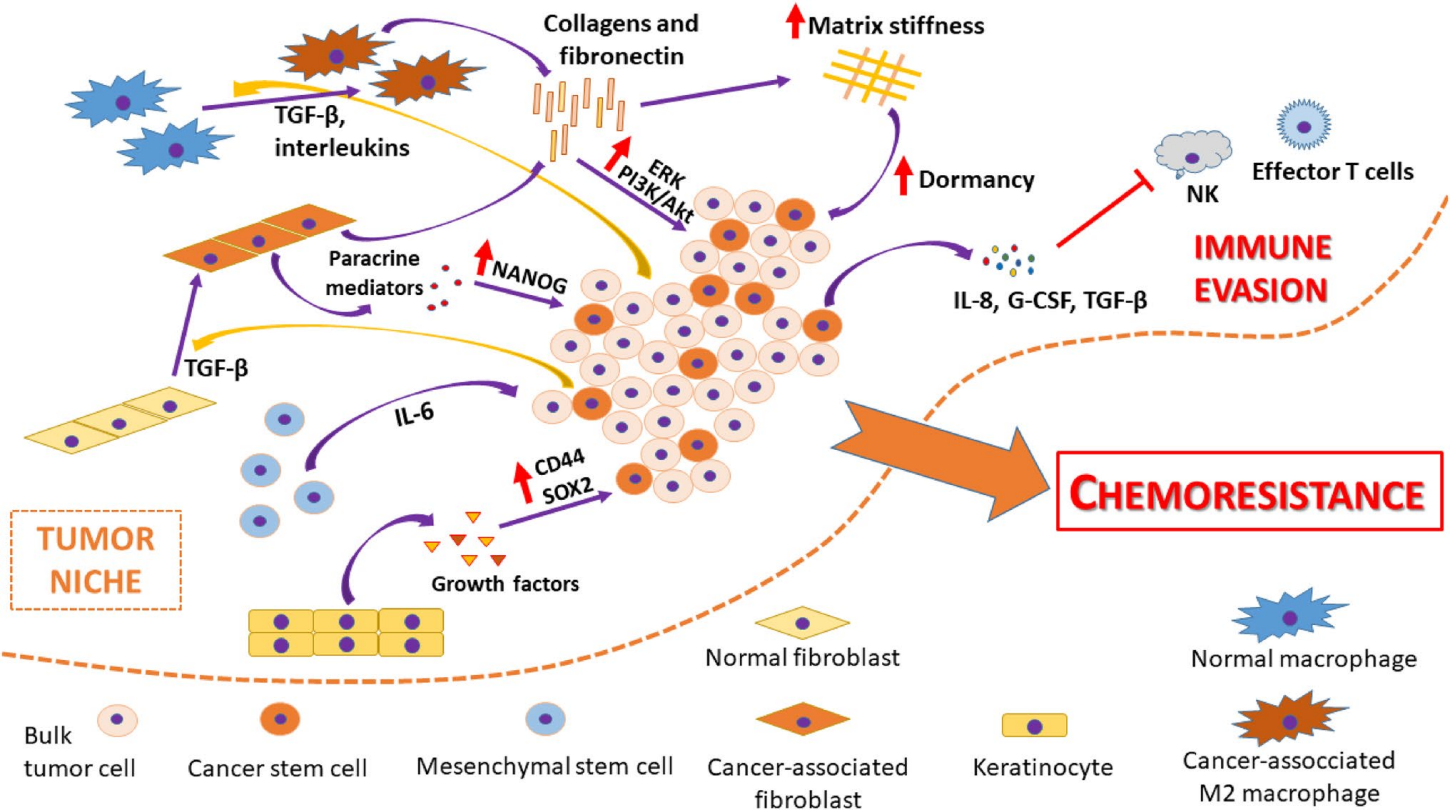
Tumor microenvironment niche. Fibroblasts, macrophages and mesenchymal stem cells infiltrate tumors and are hijacked by cancer cells to support tumor survival and growth by promoting stemness, dormancy, immunoediting, and by altering the structure of the extracellular matrix. All these result in chemoresistance (Dianat-Moghadam et al. 2022, 2023; Dzobo 2020; Dzobo et al. 2023; Gupta et al. 2021; Jingyuan et al. 2023; Kok 2020; Senthebane et al. 2018, 2017)

Solid tumors consist of multiple cell types, and recent evidence points to the crucial role of alterations in TME for epithelial neoplastic transformation (White and Lowry 2015). Apart from the heterogenous clones of neoplastic cells (both bulk and stem-like cells), non-neoplastic cells, including fibroblasts, macrophages, mesenchymal stem cells, endothelial cells and immune cells, are also present in TME (Dzobo 2020; Dzobo et al. 2023; Kok 2020). These stromal cells infiltrate the tumor and become hijacked by cancer cells to support tumor growth and drug resistance, thus pointing to the inhibition of tumor-stroma interactions as a key target in chemosensitization (Senthebane et al. 2017). The interaction between cancer cells and stromal cells is multidirectional, and cancer stem cells continuously interact with these cells to establish a favorable niche (Fig. 2) (Dianat-Moghadam et al. 2023; Huang et al. 2020a, b). Tumor-associated macrophages (TAMs) increased CSC fraction by elevating the level of hyaluronic acid in ECM, and subsequent stimulation of CD44/PI3K pathway (Gomez et al. 2020). The significance of such intercellular cross-talk in drug resistance acquisition may be indirectly confirmed by an observation that stronger infiltration of HNSCC tumors with TAMs predicted worse response to chemoradiotherapy and was associated with higher risk of relapse (Balermpas et al. 2014).

Most stromal cells are able to secrete pro-tumorigenic factors, including growth factors (e.g., TGF-beta) or cytokines and chemokines, which promote survival, stemness and chemoradioresistance (Dzobo et al. 2023; Senthebane et al. 2017). In this regard, patient-derived cancer-associated fibroblasts (CAFs) have been shown to possess the ability to promote cisplatin resistance in cancer cells by paracrine effects. Changes in gene expression, including NANOG upregulation, mediated these effects (Peltanova et al. 2021). A recent study found that these cells were able to induce a transition of SCC-25 cells into paclitaxel-resistant cells by the paracrine action of IL-6 (Liu et al. 2021a, b). Also, “normal” keratinocytes present in the cancer field can contribute to stemness and chemoresistance induction. One of the mechanisms involved relies on the secretion of ligands that activate EGFR and/or FGFR receptors on cancer cells and promote the enrichment of CD44^high^SOX2^high^ cells. This results in enhanced resistance to small molecule PI3K inhibition, which can be abolished by erlotinib (Nguyen et al. 2022). Thus, drug resistance cannot be considered as the property of isolated cancer cells but of the cell interactome characteristic of the tumor microenvironment (Dzobo et al. 2020). In addition, CAFs and TAMs are responsible for the increased production of the components of the extracellular matrix (ECM), which can contribute to chemoresistance (Dzobo et al. 2023; Senthebane et al. 2018). Indeed, ECM activated ERK and PI3K/Akt signaling in cancer cells and reduced sensitivity to cisplatin, fluorouracil and epirubicin, and the reduction in the level of collagen type I and fibronectin in ECM resulted in diminished colony formation of cancer cells and sensitization to cisplatin (Senthebane et al. 2018). Moreover, increased matrix stiffness, which was caused by increased deposition of fibrillar collagens and other proteins, together with enhanced matrix cross-linking, led to the stimulation of cell dormancy, and correlated with shorter relapse-free survival (Jingyuan et al. 2023).

The tumor microenvironment in solid tumors significantly contributes to immunosuppression (Dianat-Moghadam et al. 2022, 2023). Thus, despite the high prevalence of PD-L1 expression in HNSCC, the immune checkpoint inhibitors do not show satisfactory clinical response due to primary and adaptive resistance, including the immunosuppressive capabilities presented by CD44 + cells (Kok 2020). Cancer stem cells can affect immune cells by exosomes (Gonzalez-Callejo et al. 2023). Indeed, CSC-derived exosomes can mediate communication with other cells to protect the CSC-niche and to promote relapse (Gupta et al. 2021). Furthermore, CD44 + HNSCC cells were shown to secrete increased amounts of IL-8, granulocyte colony-stimulating factor, and TGF-β, leading to the inhibition of effector T or NK cells. In addition, CD44 + cells decreased the secretion of interferon gamma or IL-2 by peripheral blood mononuclear cells (Chikamatsu et al. 2011). All these effects may contribute to immune evasion in HNSCC.

Table 2 lists the chemicals that exhibit chemosensitizing effects by affecting HNSCC stem cells. Future research should focus on characterizing the clinical utility and optimizing the proposed strategies.

**Table 2:** A list of compounds which increased chemosensitivity by targeting stem cells in HNSCC

Compound	Biological effects	References
Celastrol	Celastrol significantly reduced the viability of cisplatin-resistant cells and suppressed tumorsphere formation	Chen et al. (2020)
Cucurbitacin I	Decreased the fraction of ALDH + /CD44 + cells and improved radio sensitivity by down-regulating STAT3, leading to synergistic effects in mice and preventing metastases	Chen et al. (2010)
Curcumin	Curcumin reduced the expression of stemness markers and reversed cisplatin-induced CD44 + and SP cell ratios by RXRα inhibition; combination with cisplatin led to stronger tumor growth reduction in a mouse xenograft model	Jiang et al. (2018)
Curcumin difluorinated	Pre-treatment with liposomal CDF killed CD44^high^ cells in cisplatin-resistant cell lines	Basak et al. (2015)
Honokiol	Honokiol in combination with cisplatin was potent in reducing the number of secondary tumor spheres by inhibiting the IL-6/STAT3 pathway	Chang et al. (2018)
Magnolol	Magnolol sensitized cancer stem cells to cisplatin leading to viability reduction similar to parental cells	Peng et al. (2022)
Melatonin	Melatonin sensitized CD44^high^ cells to cisplatin-induced cell death	Shigeishi et al. (2022)
Sulforaphane	Sulforaphane sensitized CD44 + CD271 + cells to cisplatin and 5-fluorouracil by inhibiting Hedgehog pathway and reducing SOX2 and OCT4 expression	Elkashty and Tran (2020)
5-aminolevulinic acid (ALA)	Photodynamic therapy using ALA reduced the ratio of CD44 + and ALDH + cells, decreased the expression of OCT4 and NANOG, and sensitized stem-like cells to cisplatin and 5-fluorouracil	Yu and Yu (2014)
Tubastatin A	HDAC6 inhibition by tubastatin A reduced the stemness phenotype and reversed cisplatin-induced stem cell accumulation	Tavares et al. (2022)
Vorinostat, entinostat	Inhibition of histone deacetylases diminished the stem cell population from cisplatin-resistant cell lines	Lima de Oliveira et al. (2022)
Valproic acid	Reduced the proportion of CD44 + cells, sensitized cells to cisplatin treatment, and its combination with cisplatin reduced tumor burden in mice	Lee et al. (2015)
JQ1	BET proteins inhibition with JQ1 reduced the expression of IL-6/8, BMI1, and CD44, as well as diminished the growth of xenograft tumors formed by CD44^high^/ALDH^high^ cells derived from cisplatin-resistant cell lines	Dong et al. (2021)
NCT-501	Inhibition of ALDH activity by NCT-501 sensitized cisplatin-resistant CAL27 cells to cisplatin and reduced spheroid formation capacity	Kulsum et al. (2017)
Aldi-6	Inhibition of ALDH activity by Aldi-6 sensitized cells to cisplatin by increasing reactive oxygen species formation	Kim et al. (2017)
PTC-209	Bmi1 inhibition by PTC-209 sensitized tumor cells to cisplatin and reduced the ratio of Bmi1 + stem cells and reduced tumor growth and metastasis	Chen et al. (2017)
S3I-201	S3I-201 reduced the percentage of SP cells and CD44 + cells; STAT3 inhibition by S3I-201 in combination with cisplatin, 5-fluorouracil, or docetaxel reduced the number and size of tumorspheres	Bu et al. (2015)
Tocilizumab	The inhibition of the IL-6R/STAT3 pathway by tocilizumab suppressed cisplatin-induced accumulation of stem cells; combination therapy suppressed orosphere formation and decreased xenograft tumor growth	Herzog et al. (2021)
SB225002	Inhibition of IL-8 activity by SB225002 in combination with cisplatin significantly reduced the viability of CD10^high^ cells	Pu et al. (2021)
Afatinib	Pre-treatment of cells with afatinib (EGFR inhibitor) downregulated CD44 and OCT3/4 and abrogated the enrichment of SP cells induced by ionizing radiation, increasing radiosensitivity	Macha et al. (2017)
GDC0449	Hedgehog pathway inhibition with GDC0449 sensitized CD10^high^ cell tumors to cisplatin	Wang et al. (2021)
XAV939	Inhibition of the Wnt/β-catenin pathway with XAV939 reversed cisplatin resistance and reduced the proportion of SP cells	Sinnung et al. (2021)
	XAV939 sensitized cells to cisplatin and reduced the expression of stem cell markers (CD44, KLF4, OCT4, and β-catenin)	Roy et al. (2019)
Ibrutinib	Ibrutinib (BTK inhibitor) reduced the expression of CD133 and NANOG, and the percentage of ALDH-positive cells; it sensitized cells to cisplatin, and the combination of cisplatin and ibrutinib significantly reduced tumorsphere formation	Liu et al. (2021a, b)
U0126	MEK/ERK inhibition with U0126 in combination with cisplatin significantly reduced the viability of CD44^high^ cells	Huang et al. (2020a, b)
SB203580	p38 inhibition by pre-treatment with SB203580 sensitized cells to cisplatin and attenuated cisplatin-induced expression of stem cell markers (SOX2, OCT4, CD44)	Roy et al. (2018)
Temsirolimus	Temsirolimus reduced the fraction of CD44^high^/ALDH^high^ cells and sensitized cells to cisplatin; pre-treatment with temsirolimus was the best strategy for reducing CSC fraction upon cisplatin treatment	Nakano et al. (2021)
ABT-199	Bcl-2 inhibition using ABT-199 diminished the proliferation, migration and invasion of CD44 + /ALDH + SP cells derived from SQ20B laryngeal cancer cell line; ABT-199 synergized with cetuximab in reducing tumor volume in vivo	Guy et al. (2021)

*SP* side population cells

## Conclusions

The current state of knowledge allows us to assume that HNSCC cancer stem cells are the most significant population of cells in the acquisition of drug resistance. Importantly, the stem cell hypothesis does not fully explain the occurrence of chemoresistance (Griso et al. 2022). For example, it has been shown that the pH reduction in tumors is responsible for cisplatin resistance because of drug entrapment in the acidic extracellular compartment. While microenvironment acidification could induce the expression of NANOG, CD44, and BMI1 in cells in vitro, it was shown that the restoration of physiological pH was sufficient for cell resensitization to cisplatin (de Bem Prunes et al. 2022). There is also a plethora of other mechanisms which can contribute to the resistant phenotype, but not all of them have been studied in relation to HNSCC stem cells (Griso et al. 2022).

Nevertheless, based on the current literature, the association between the stemness phenotype and chemoresistance in HNSCC is evident. Crucial aspects of this connection include the finding that chemotherapy leads to the enrichment of cancer stem cells (Basak et al. 2015; Bu et al. 2015; Jiang et al. 2018; Kim et al. 2017; Nakano et al. 2021; Nör et al. 2014; Subramanian et al. 2017, p. 90), drug-resistant cells show the enrichment of stem cell subpopulations (Kulsum et al. 2017; Lima de Oliveira et al. 2022; Murakami et al. 2022; Roy et al. 2018, 2019; Tsai et al. 2011), and isolated cancer stem cells are resistant to chemotherapy (Chen et al. 2010; Fernandes et al. 2022; Gunduz et al. 2019; Silva Galbiatti-Dias et al. 2018). In addition, various molecular mechanisms underlying stemness-related therapy resistance were identified (Barbato et al. 2019). The PI3K/Akt, Wnt/catenin, and Src pathways are all implicated, as are interleukin-induced STAT3 activation, epigenetic modulators, redox states, and the tumor microenvironment. Also metabolic reprogramming, one of the hallmarks of cancer, can contribute to stemness and chemoresistance. The activity of the enzymes associated with NAD + synthesis and consumption is frequently altered in cancer cells, including head and neck cancers (Togni et al. 2021). These aberrations may play a role in the acquisition of stemness potential (Novak Kujundžić et al. 2021). A recent study has shown that NAD + imbalance is characteristic of HNSCC stem cells. The targeting of NAD + biosynthetic pathways with the inhibitors of nicotinamide phosphoribosyltransferase (NAMPT) or nicotinate phosphoribosyltransferase (NAPRT) showed anti-tumor effects and exerted sensitization to docetaxel in xenograft mice. Moreover, the adaptive reboosting of NAD + synthesis by the upregulation of NAMPT or NAPRT, which was observed upon cell treatment, could be tackled by the combinatorial inhibition of both enzymes (Navas et al. 2023). This corroborates the importance of the use of mixes of chemicals to deal with the consequences of cell plasticity. Additionally, the isoenzymes of pyruvate dehydrogenase kinase (PDK1 and PDK2), which are associated with alterations of glucose metabolism called the Warburg effect, have been implicated in stemness and chemoresistance. Their knockdown led to the HNSCC sensitization to cisplatin and gemcitabine (Sun et al. 2022a, b). Interestingly, recent reports presented a new strategy for eradicating HNSCC stem cells by inducing their osteogenic differentiation (Jaksic Karisik et al. 2023; Patil et al. 2022).

Thus, there is a plethora of biological mechanisms responsible for stemness-induced chemoresistance and because of this very reason it is currently difficult to single out a target which would be best for the effective sensitization of stem cells in tumors (Yang et al. 2020). Perhaps, this would require some personalization using molecular diagnostic tests that have not yet been developed (Walcher et al. 2020). However, the difficulty in the selection of drug targets is also a consequence of high cellular plasticity (Salem and Salo 2023). Indeed, isolated subpopulations of stem cells were able to restore the original heterogenous cell populations (Navas et al. 2023). Thus, combinatorial sensitizing treatments may be the best option; however, more work is necessary to determine which compounds and targets show the highest synergistic potential. Furthermore, most of the currently available information was developed using selected cell lines and more research should be peformed in vivo, especially using patient-derived xenografts (or patient-derived organoids) or other relevant in vivo models (Salem and Salo 2023). Moreover, most in vivo studies used tumor size/volume as endpoint, but rarely analyzed cell subpopulations in the tumors, which would be helpful to prove that the observed effects are indeed dependent on the ablation of stem cells. Another factor that needs further elucidation is the sequence of treatments. Some evidence points to the utility of sequential treatments, with the stem cell-ablating chemical preceding the classical chemotheraputic dug. However, more evidence is necessary to find the best option. Thus, while the benefits of the pharmacological targeting of cancer stem cells by affecting various molecular targets were shown in vitro and in vivo, direct evidence of such benefits in HNSCC patients have not been documented so far, and the field needs well-designed relevant clinical studies which would test the clinical validity of the findings.

This paper corroborates targeting cancer stem cells as a promising strategy for overcoming therapy resistance in head and neck cancers. Chemicals aimed at stem cell ablation have shown adjuvant potential in animal studies, which warrants further research on the exact clinical utility of these stem cell-targeted strategies as chemosensitizers in humans.

## Data Availability

Data sharing not applicable to this article as no datasets were generated or analysed during the current study.
